# Bubble-enhanced cold plasma improves microbial inactivation in water under scalable conditions

**DOI:** 10.1007/s00253-026-13979-8

**Published:** 2026-08-03

**Authors:** José Gonçalves, João Pequeno, Jure Žigon, Tom Koritnik, Filipe Ferreira da Silva, Davor Kržišnik, Marija Trkov, Israel Diaz, Elisa Rodriguez, Sebastian Dahle, Andrej Steyer, Rita Maurício, Graça Martinho

**Affiliations:** 1 MARE - Marine and Environmental Sciences Centre, ARNET - Aquatic Research Network Associate Laboratory, NOVA School of Science and Technology, NOVA University Lisbon, Caparica, Portugal; 2 Department of Wood Science and Technology, Biotechnical Faculty, University of Ljubljana, Ljubljana, Slovenia; 3 Department for Public Health Microbiology, National Laboratory of Health, Environment and Food, Ljubljana, Slovenia; 4 CEFITEC – Centre of Physics and Technological Research, NOVA School of Science and Technology, NOVA University Lisbon, Caparica, Portugal; 5 Institute of Sustainable Processes, University of Valladolid, Paseo Prado de La Magdalena 3-5, Valladolid, 47011 Spain; 6 Department of Chemical Engineering and Environmental Technology, University of Valladolid, Paseo Prado de La Magdalena 3-5, Valladolid, 47011 Spain

**Keywords:** Cold atmospheric plasma, Environmental biotechnology, Water disinfection, Bioprocess engineering, *Escherichia coli*, *Bacillus subtilis*

## Abstract

**Abstract:**

Cold atmospheric plasma (CAP) is emerging as an environmentally friendly disinfection technology, but most plasma-liquid systems remain confined to millilitre‑scale volumes, limiting their applicability as bioprocesses for water treatment. Here, we designed and evaluated a bubble‑enhanced dielectric barrier discharge reactor, the Spiral Plasma Aeration Reactor Column (SPARC), as an environmental technology approach for litre‑scale inactivation of model bacteria in water. Direct CAP exposure achieved rapid, near‑complete inactivation of *Escherichia coli* and *Bacillus subtilis* in 200 µL, but performance decreased sharply at 100 mL, confirming strong volume dependence under surface‑limited plasma-liquid contact. Coupling CAP with gas bubbling (2 L·min^−1^) in SPARC markedly enhanced inactivation at 250–500 mL, achieving approximately 2–3 log reductions (≥ 99–99.9%) for *E. coli* and *B. subtilis* within 60 min, whereas non-aerated CAP remained below ≈0.8-log (≤ 60%) reduction under identical conditions. At 1 L under high initial loads (~ 10^6^–10^7^ CFU·100 mL^−1^), bubble-assisted CAP yielded ~ 4-log inactivation of *E. coli* and 3–3.5-log reduction of *B. subtilis*, including continued post-treatment inactivation consistent with plasma-activated water effects. In a protein‑rich organic matrix, early‑stage inactivation was attenuated, but SPARC still achieved ≈2–3‑log reductions, indicating robust yet matrix‑sensitive performance under wastewater‑like conditions. At fixed electrical input (34.5 W), specific energy consumption ranged from 60 kWh·m^−3^ (250 mL, 30 min, > 99% *E. coli* inactivation) to 30 kWh·m^−3^ (1 L, 60 min, ≈4‑log *E. coli* inactivation), corresponding to ≈7.5 kWh·log₁₀^−1^·m^−3^. These results position bubble‑assisted CAP as a promising environmental biotechnology and bioprocess platform for water disinfection and emphasise the central role of reactor and contactor design in determining microbial efficacy and energy demand.

**Key points:**

• *Direct CAP exposure in microlitre-scale water (200 µL) produces rapid ≥ 3-log₁₀ (> 99.9%) inactivation of E. coli and B. subtilis, but performance deteriorates at 100 mL, confirming strong volume dependence under surface-limited plasma-liquid contact.*

• *The bubble-enhanced DBD reactor SPARC, operated at 250–500 mL, achieves ≈2–3-log₁₀ (≥ 99–99.9%) bacterial reductions within 60 min, whereas non-aerated CAP under identical electrical conditions generally remains below ≈0.8-log₁₀ (≤ 60%) reduction.*

• *At 1 L, bubble-assisted SPARC attains ~ 4-log₁₀ (E. coli) and 3–3.5-log₁₀ (B. subtilis) inactivation under high initial loads, with specific energy consumption between 30 and 60 kWh·m*^*−3*^* (≈7.5 kWh·log₁₀*^*−1*^*·m*^*−3*^*) for the laboratory prototype.*

## Introduction

The dissemination of antibiotic-resistant bacteria (ARB) and antibiotic resistance genes (ARGs) in aquatic environments has emerged as a critical environmental issue, posing significant risks to public health and threatening the ecological balance of receiving water bodies (Yang et al. 2020; Gonçalves et al. 2023; Ho et al. 2025). ARB and ARGs are widely detected in aquatic ecosystems, wastewater, and receiving environments (Drummond et al. 2023; Rosa et al. 2025). Wastewater treatment plants (WWTPs) are recognized as hotspots for selective pressure and horizontal gene transfer (HGT), where high microbial densities and diversity, residual antibiotics, and other stressors promote the persistence and dissemination of ARB and ARGs (Gonçalves et al. 2023; Punch et al. 2025).

Ensuring microbial safety in treated wastewater is a growing challenge, especially as treatment processes advance to tertiary and quaternary stages (Adeel et al. 2024; Solcova et al. 2024; Silvester et al. 2025). Traditional disinfection methods (e.g. chlorination and ultra-violet (UV) irradiation) have limitations that drive the search for alternative technologies, including the formation of disinfection by-products, reduced efficacy in the presence of organic matter or particle shielding, the absence of residual antimicrobial activity in UV-based systems, and limited effectiveness against some microorganisms (Gonçalves et al. 2025; Kuddushi et al. 2025; Pequeno et al. 2026). In recent years, non-thermal plasma, commonly referred to as cold atmospheric plasma (CAP), has emerged as a promising approach for water disinfection due to its ability to generate reactive species in situ and effectively inactivate a broad range of waterborne pathogens (Gonçalves et al. 2025; Lu et al. 2025; Pequeno et al. 2026). CAP produces a complex mixture of reactive oxygen and nitrogen species (RONS), together with UV photons and transient electric fields, which act synergistically to damage bacterial cell membranes, nucleic acids, and proteins (Lunder et al. 2025). Importantly, microbial inactivation by CAP occurs rapidly and without the formation of persistent or harmful residuals, positioning CAP as a cleaner and potentially sustainable alternative to conventional chemical disinfectants (Das et al. 2022; Ahmed et al. 2025).

CAP has demonstrated high efficacy against a wide range of microorganisms, including bacteria, viruses, and even highly resistant fungal spores (Qin et al. 2024; Helmke et al. 2011; Ji et al. 2018). Near-complete inactivation of *Escherichia coli* in water has been reported using dielectric barrier discharge (DBD) CAP systems, achieving multi-log reductions within 20 min of treatment (Triantaphyllidou and Aggelopoulos 2025). CAP has also proven effective against particularly challenging microorganisms, such as *Listeria monocytogenes* (Lu et al. 2014) and *Salmonella enterica* (Ziuzina et al. 2015b), as well as complex microbial biofilms associated with human infections (Ziuzina et al. 2015a).

In addition, CAP-based treatments have been shown to inactivate fungal spores and reduce mycotoxin contamination, further highlighting the broad antimicrobial spectrum of this technology (Ma and Jiao 2022; Kuddushi et al. 2025). CAP has demonstrated strong antiviral activity in aqueous systems, including the inactivation of viruses, primarily through oxidative damage to viral envelopes and genomic material (Thirumdas 2022; Hamza et al. 2023a; Thomas et al. 2025; Thakulla et al. 2025).

However, most CAP‑based water disinfection studies remain limited to bench‑scale configurations treating small volumes (often only millilitres), and performance frequently deteriorates as treated volume increases (Morenas et al. 2025; Pequeno et al. 2026). This is largely attributed to restricted plasma-liquid interfacial area and diffusion‑limited delivery of reactive species into the bulk, which constrain microbial exposure in larger systems (Patange et al. 2019; Foster 2017; Rabinovich et al. 2022). Recent work has begun to address these constraints using multi‑discharge reactors, bubble‑enhanced CAP, and plasma activated water (PAW) recirculation, but quantitative data on microbial inactivation at sub‑litre to litre scales remain scarce (Patange et al. 2019; Rabinovich et al. 2022).

The recent pilot-scale studies achieved good contaminant and pathogen removal (often > 80–90%) (Morenas et al. 2025). Addressing these scale-up challenges will require engineering innovations (e.g. improved plasma-liquid contact through multi-discharge reactors or enhanced mixing) and further pilot studies to demonstrate that CAP can maintain rapid, uniform disinfection at larger throughputs. A few recent studies have begun to address larger‑volume CP wastewater treatment. Combined cavitation–plasma (CaviPlasma) systems have treated up to 2.5 L and beyond (Palíková et al. 2025). Cold plasma has also been applied to aquaculture water at volumes on the order of 510 mL (Balazinski et al. 2025).

Accordingly, this study aimed to develop and characterize a bubble‑enhanced CAP bioprocess for bacterial inactivation in water at sub‑litre and litre scales. Using *E. coli* and *B. subtilis* as model Gram‑negative and Gram‑positive bacteria, respectively, we systematically compared direct (surface‑contact) and bubble‑assisted DBD configurations across increasing treatment volumes (200 µL to 1 L). We quantified inactivation kinetics under standardized operating conditions, examined the influence of high initial loads and a protein‑rich wastewater surrogate matrix, and calculated specific energy consumption per volume and per log reduction. By explicitly linking reactor design, plasma-liquid mass transfer, and microbial responses, this work contributes to the environmental biotechnology and bioprocess engineering of plasma‑based water disinfection systems. The novelty of this work lies in the integrated bioprocess design and evaluation of a bubble‑enhanced DBD reactor at sub‑litre to litre scales, combining systematic volume scaling, high‑load and organic‑matrix challenges with quantitative energy‑per‑log metrics under fixed electrical input.

## Methods

### Construction of DBD CAP systems

The primary objective of the present study was to design and evaluate scalable CAP reactors for bacterial inactivation in water, with emphasis on identifying configurations capable of maintaining stable plasma operation and effective plasma-liquid interactions as treated volumes increase. To support this objective, multiple reactor design and operational parameters were systematically explored during the development phase, including dielectric barrier materials, electrode materials and geometries, tube dimensions, and the resulting inter‑electrode distances, all of which are known to influence plasma stability, electrical behaviour, and thermal performance. Several dielectric materials (polyvinyl chloride (PVC), borosilicate glass, and quartz) and electrode configurations (including rod‑ and screw‑type inner electrodes, as well as linear, fabric‑like, and helical outer electrodes) were initially tested to identify configurations compatible with sustained plasma generation under conditions relevant to scale‑up.

Two reactor configurations were selected for subsequent experiments. For direct CAP exposure at micro‑ to sub‑litre scale, a planar DBD was used, consisting of two stainless‑steel disk electrodes separated by a dielectric barrier (glass Petri plate), with liquid samples positioned directly beneath the dielectric surface for 200 µL and 100 mL assays. For bubble‑assisted CAP, a tubular DBD reactor (Spiral Plasma Aeration Reactor Column, SPARC) was employed, comprising a quartz dielectric tube with a centred stainless‑steel rod inner electrode and a spiral copper outer electrode coupled to a vertical water column and air diffuser. Both configurations exhibited consistent CAP generation, low variability in operating current, and minimal system heating during extended operation, and reactor temperature was monitored using an infrared thermometer at four key locations, including regions adjacent to the electrodes, to verify non‑thermal operating conditions and exclude heat‑driven effects.

### Plasma generation and experimental setups

Atmospheric air cold plasma was generated using a DBD-based system operated under ambient conditions. The discharge was powered by a high-voltage (HV) alternating current power supply operated at fixed settings throughout all experiments to ensure comparability between experimental conditions: The voltage was set to 15 kV, and the alternating current frequency was set to 5 kHz. The plasma source was operated in two distinct configurations: (i) direct plasma exposure, in which the discharge was applied directly above the liquid surface, and (ii) bubble-assisted plasma exposure, in which plasma-generated reactive species were transferred into the liquid via gas bubbling.

For direct CAP exposure, experiments were conducted using 200 µL and 100 mL of sterile distilled water (conductivity: 0.5–2.0 µS/cm; pH: 5.36–7.1; room temperature) placed in open containers positioned directly beneath the plasma discharge. The distance between the plasma source and the liquid surface was kept constant (0.5 cm) across all direct-exposure experiments. Plasma exposure times ranged from seconds (up to 30 s) for micro-volume experiments to several minutes (up to 600 s) for 100 mL experiments.

For bubble-assisted exposure, CAP was generated in the gas phase and introduced into the liquid through an air diffuser (JBL ProSilent Aeras Micro Ball L, diameter 40 mm, extra fine-pored quartz sand mixture) positioned at the bottom of a vertical cylindrical water column (internal diameter 8.35 cm, height 56.5 cm), producing fine bubbles and enabling indirect contact between plasma-derived reactive species and the liquid phase. A sampling port with a manual valve was installed 5 cm above the column bottom to allow sample collection. The dielectric plasma reactor tube (quartz) had a length of 24.5 cm, an inner diameter of 1.6 cm, and an outer diameter of 2.0 cm, with a centred stainless-steel inner electrode (1.0 cm diameter) and a spiral copper tape outer electrode wrapped around the external surface (Fig. 4).

Experiments were performed using 250 mL, 500 mL, and 1000 mL of sterile distilled water. Gas flow rates between 1 and 5 L·min^−1^ were evaluated during pH optimization experiments, while a flow rate of 2 L·min^−1^ was selected for all microbiological assays based on pH stabilization and plasma stability. CAP treatment time for indirect exposure experiments was fixed at 30 min, corresponding to the period after which pH values reached a stable plateau. Gas flow rates were measured and adjusted with a rotameter. For the experiments with 250 mL of distilled water, pH was measured using a pH probe (pHenomenal® pH Electrodes, VWR, USA), and oxidation–reduction potential (ORP) was measured using an IntelliCAL™ MTC101/MTC30 ORP electrode (Hach, USA).

For the experiments performed with 250 mL of sterile distilled water at a gas flow rate of 2 L·min^−1^, the concentrations of nitrite, nitrate, hydrogen peroxide, and hydroxyl radicals were quantified using spectrophotometric and fluorometric assays. Briefly, nitrite (NO₂⁻) was measured with the Griess reagent method: 500 µL of sample was mixed with 500 µL of Griess reagent, incubated for 15 min at room temperature, and the absorbance was recorded at 540 nm using a UV–Vis spectrophotometer (Thermo Scientific, USA). Nitrite concentrations were obtained from calibration curves constructed with sodium nitrite (NaNO₂) standards of known concentration. For nitrate (NO₃⁻), 4 drops of 1 M hydrochloric acid (HCl) were added to 2 mL of sample, and the UV–Vis spectrum was acquired; the corrected absorbance was calculated by subtracting twice the absorbance at 275 nm from the absorbance at 220 nm, and nitrate concentrations were determined using calibration curves prepared from sodium nitrate (NaNO₃) standards. Hydrogen peroxide (H₂O₂) was quantified by adding 100 µL of titanium(IV) oxysulfate–sulfuric acid (TiOSO₄) reagent to 1 mL of sample, allowing the reaction to proceed for 10 min to form a yellow pertitanic acid complex, and measuring the absorbance at 403 nm with a UV–Vis spectrophotometer (Thermo Scientific, USA). H₂O₂ concentrations were calculated from calibration curves generated with hydrogen peroxide standard solution. Hydroxyl radicals (⋅OH) were quantified using terephthalic acid (TPA) as a fluorescent probe. A solution containing 2.5 mM NaOH and 0.2 mM TPA was prepared in water and exposed to plasma under the same 250 mL, 2 L·min^−1^ conditions. Samples collected at defined time points were analyzed using a fluorescence spectrometer (Cary Eclipse, Agilent Technologies, USA), with excitation at 310 nm and emission at 423 nm, and ⋅OH concentrations were determined by correlating the fluorescence intensity with calibration curves obtained from 2-hydroxyterephthalic acid (hTPA) standard solutions.

Control experiments were conducted under identical conditions (same period of plasma on) but without the bubble diffuser, allowing direct comparison between CAP treatment with and without enhanced gas–liquid mass transfer. For the high-load experiments, the same conditions were used, except the plasma was applied for 60 min followed by a 30 min off period (no plasma) to observe any post-treatment effect.

To assess the performance in a complex organic matrix representative of challenging water quality conditions, an additional experiment was conducted using a reconstituted meat stock solution prepared in 1000 mL of sterile distilled water by adding and boiling four meat stock cubes according to the manufacturer’s instructions (1 cube per 250 mL; Knorr). The stock matrix was used as a wastewater-relevant challenge medium due to its elevated organic content and ionic strength relative to distilled water, which can increase oxidant demand and influence plasma-derived reactive species availability. Electrical power input was measured at 34.5 W (15 kV, 2.3 mA) using the high-voltage supply readout. The high-voltage power supply for plasma discharge ignition is a home-build design, using a 24 V, 10 A switch-mode power supply (Joylit S-240-24) in combination with a programmable DC/DC voltage converter (Joy-it DPS5015) to drive a commercial high-voltage module (ZVS_Driver_20A_kit_AC, Voltagezone Electronics e.U., Graz, Austria) based on the Mazilli zero-voltage switching driver design (Hapidin et al. 2017) with a non-rectifying flyback transformer capable of outputting up to 15 kV AC HV. The plasma was generated with the input voltage of the HV module set to 20 V.

Specific energy consumption was calculated as $E=\frac{P\cdot t}{V}$ (kWh·m^−3^), where P is the electrical power supplied to the high‑voltage module (kW), t is treatment time (h), and V is volume (m^3^). The gas phase was supplied from compressed air, and no air pumps were operated.

### Bacterial strains and preparation

*Escherichia coli* (DH5α) and *Bacillus subtilis* (L12) (Fisher Scientific, USA) were used as model Gram-negative and Gram-positive bacteria, respectively. Bacterial strains were cultivated aerobically in Miller’s LB Broth Base (Invitrogen, USA), prepared according to the manufacturer’s instructions, and incubated at 37 °C overnight. Prior to each experiment, cultures were subcultured three successive times to ensure physiological consistency. To standardize the initial bacterial concentrations across experiments, calibration curves correlating optical density (OD_600_) with colony-forming units (CFU) were established using LB agar (Lennox formulation; Merck, Germany).

Cultures were initiated from a single colony inoculated into LB broth and incubated overnight. This procedure was repeated three times. The bacterial cells were then washed by centrifugation at 4000 rpm using a Micro Star 12 centrifuge (VWR, USA) with phosphate-buffered saline (PBS; Merck, Germany) to remove residual growth medium. The resulting pellets were resuspended in sterile distilled water and adjusted to the desired initial concentrations prior to plasma treatment.

Bacterial suspensions were freshly prepared for each experimental replicate, and water samples were inoculated immediately before plasma exposure to minimize changes in bacterial viability unrelated to plasma treatment. Between biological replicates, the reactor and all wetted components were thoroughly cleaned to prevent cross-contamination. The system was flushed with sterile distilled water under continuous flow for 1 h prior to each new experiment. To verify the effectiveness of the cleaning procedure, control samples of the flushed water were collected and plated following the same culture-based enumeration protocol used for experimental samples. No bacterial growth was detected in any control plates, confirming the absence of residual contamination between experimental runs*.*

### Microbial enumeration and data analysis

Bacterial inactivation was quantified using standard culture-based enumeration. At predefined time points during plasma exposure, liquid samples were collected aseptically and immediately serially diluted in PBS buffer. Serial dilutions ranged from 10^0^ to 10^−8^, depending on the expected bacterial concentration. Aliquots of appropriate dilutions were plated in triplicate on solid growth media and incubated at 37 °C for 24 h prior to colony counting. Results were expressed as colony-forming units (CFU) per 100 mL. The detection limit of the method corresponded to the lowest dilution plated. All experiments were performed with three independent biological replicates (*n* = 3), each obtained from a different bacterial culture. Data are presented as mean values ± standard deviation. Log₁₀ reduction was calculated as ${\mathrm{l}\mathrm{o}\mathrm{g}}_{10}({N}_{0})-{\mathrm{l}\mathrm{o}\mathrm{g}}_{10}({N}_{t})$, where ${N}_{0}$ and ${N}_{\mathrm{t}}$ are the initial and time—*t* CFU·100 mL^−1^, respectively. Percentage reduction was calculated as $(1-{N}_{\mathrm{t}}/{N}_{0})\times 100\mathrm{\%}$.

## Results

**Fig. 1:**
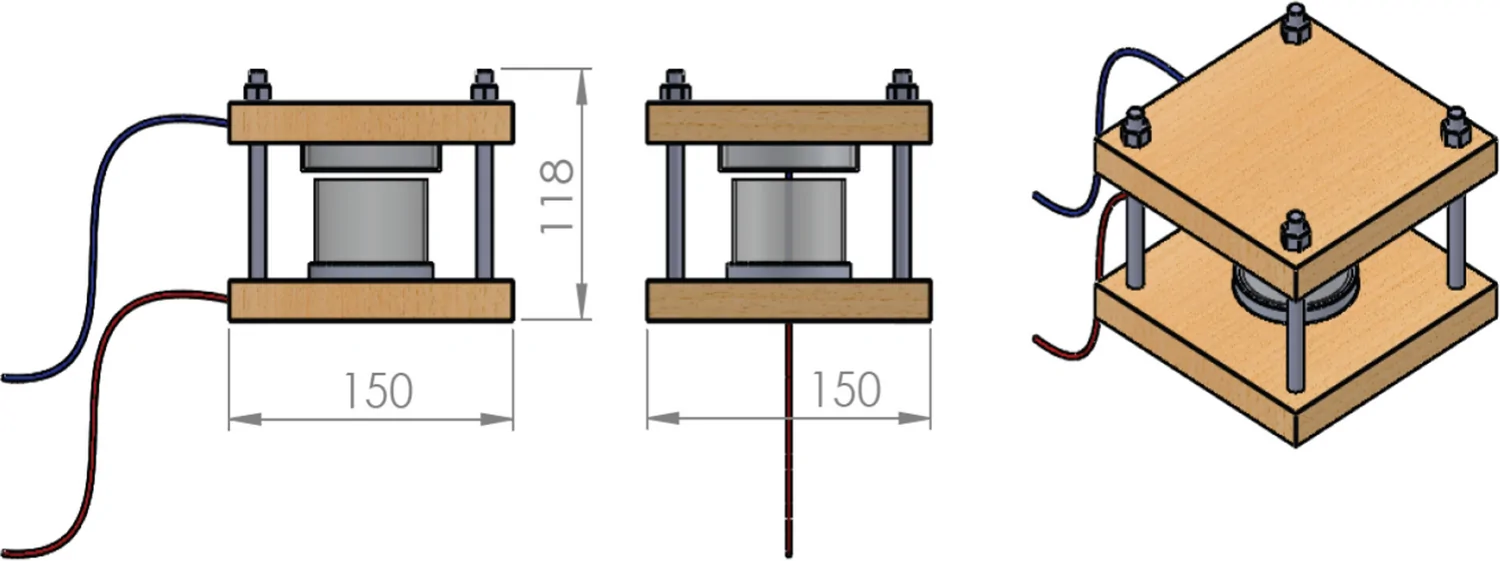
Schematic of the direct CAP treatment set-up. The reactor consists of two stainless-steel disk electrodes separated by a dielectric barrier (glass Petri plate) forming a planar DBD, mounted in a mechanical frame with a sample opening whose height is adjustable up to 118 mm. This adjustable opening allows the liquid container to be positioned directly beneath the dielectric barrier according to the treated volume (in this study, 200 µL and 100 mL)

**Fig. 2:**
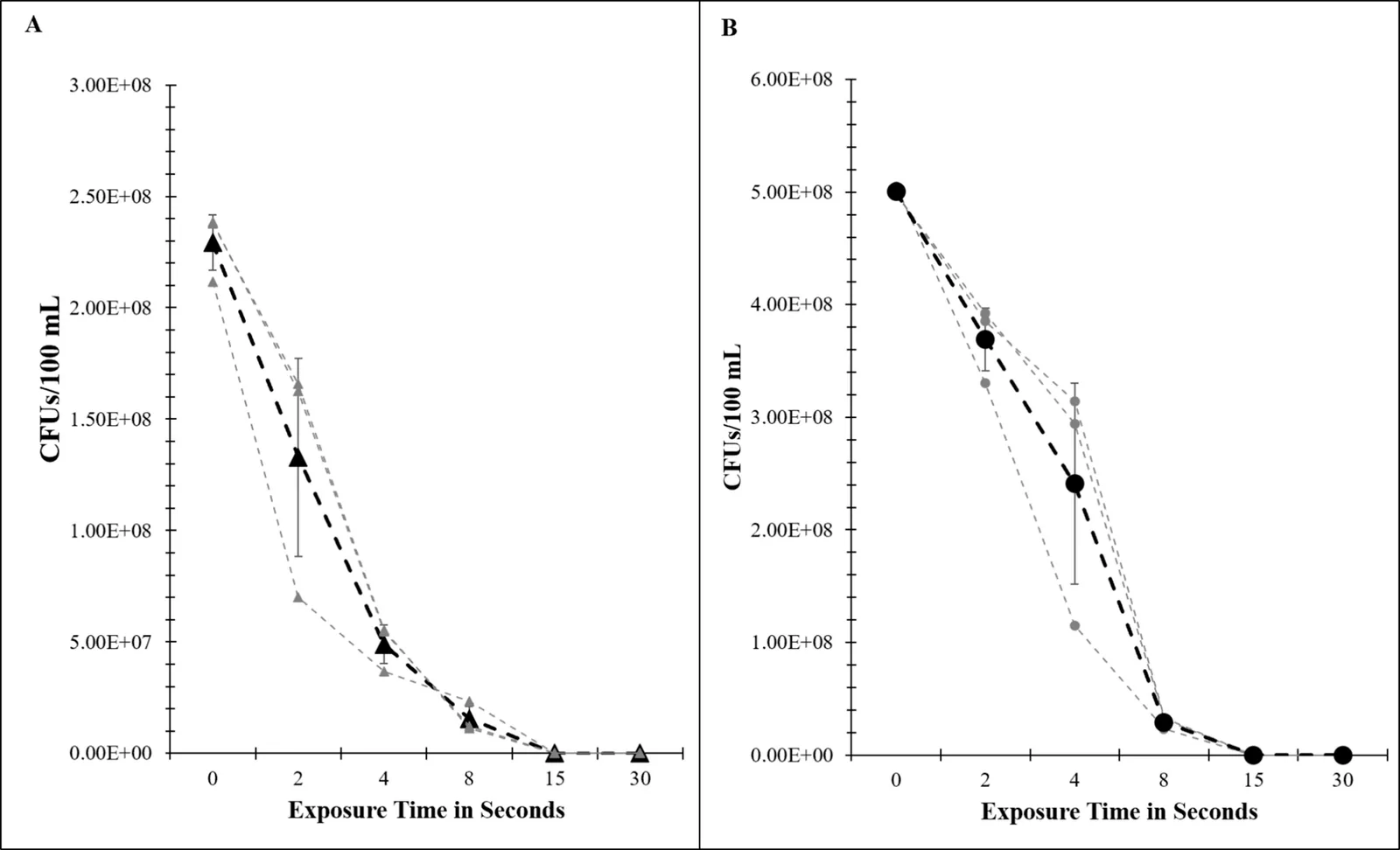
Inactivation of model bacteria in 200 µL of water. **A** *Escherichia coli* and **B** *Bacillus subtilis* survival as a function of direct CAP exposure time. Individual dashed lines represent independent biological replicates (*n* = 3), while the dash black line with symbols indicates the average CFU concentration. Error bars show the standard deviation of replicates

Direct CAP exposure experiments were first performed under surface plasma-liquid contact conditions to establish baseline inactivation performance as schematized in Fig. 1. In these assays, 200 µL of sterile distilled water inoculated with bacteria was directly exposed to CAP, and viable counts were quantified by culture-based enumeration using serial dilutions from 10^0^ to 10^−8^. Direct exposure produced a rapid, time‑dependent decrease in viable counts for both model organisms (Fig. 2). For *E. coli* (Fig. 2A), pronounced inactivation occurred within the first seconds of CAP exposure, with approximately 0.6‑ and 1.5‑log₁₀ reductions (73.5% and 96.9%) after 4 and 8 s, respectively. No viable CFU were detected at 15 and 30 s, indicating a nearly complete inactivation within the detection limits of the culture-based method. *B. subtilis* (Fig. 2B) showed a slower inactivation profile under the same conditions, with about 0.1‑ and 1.2‑log₁₀ reductions (26.2% and 94.2%) after 8 s. As observed for *E. coli*, no culturable *B. subtilis* cells were detected after 15 and 30 s of treatment.

**Fig. 3:**
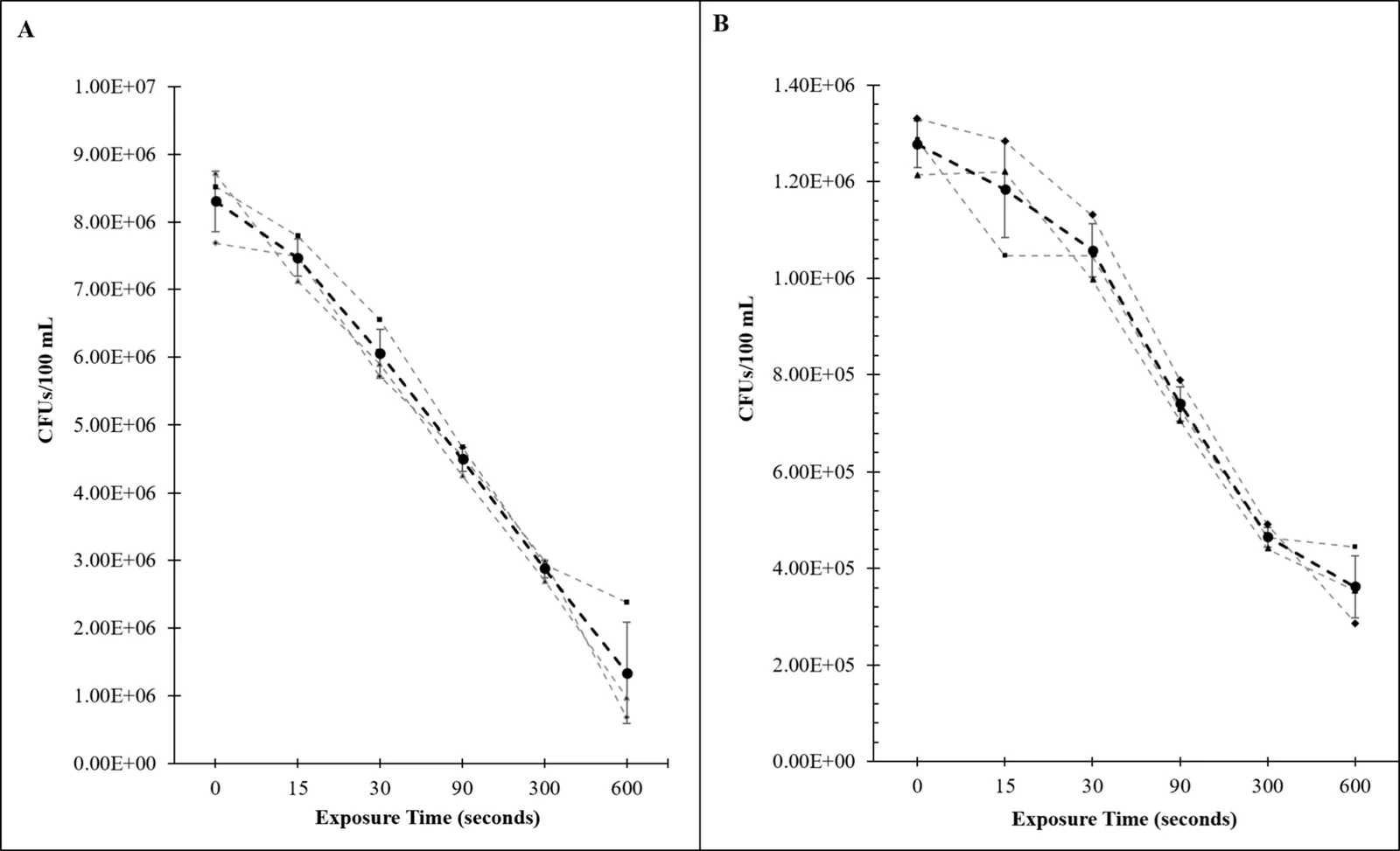
Inactivation of model bacteria in 100 mL of water. **A** *Escherichia coli* and **B** *Bacillus subtilis* survival as a function of CAP exposure time. Individual dashed lines represent independent biological replicates (*n* = 3), while the dash black line with symbols indicates the mean CFU concentration. Error bars represent the standard deviation of replicates. Results are expressed as CFU per 100 mL

To evaluate whether the high inactivation efficiencies observed at micro-scale could be maintained at larger treated volumes, the same CAP configuration was applied to 100 mL of water (Fig. 3). In this configuration, inactivation proceeded more gradually and required substantially longer exposure times. For *E. coli* (Fig. 3A), mean concentrations decreased from 8.31 × 10^6^ CFU·100 mL^−1^ at time zero to 6.05 × 10^6^ CFU·100 mL^−1^ after 30 s and 1.34 × 10^6^ CFU·100 mL^−1^ after 600 s of CAP treatment, corresponding to approximately 0.3‑ and 0.8‑log₁₀ reductions (45.9% and 83.9%), respectively. For *B. subtilis* (Fig. 3B), inactivation was slower and more variable at early time points, with mean CFU values decreasing from 1.28 × 10^6^ CFU·100 mL^−1^ initially to 3.61 × 10^5^ CFU·100 mL^−1^ after 600 s, equivalent to approximatly 0.2- and 0.6-log₁₀ reductions (42.1% and 71.7%).

**Fig. 4:**
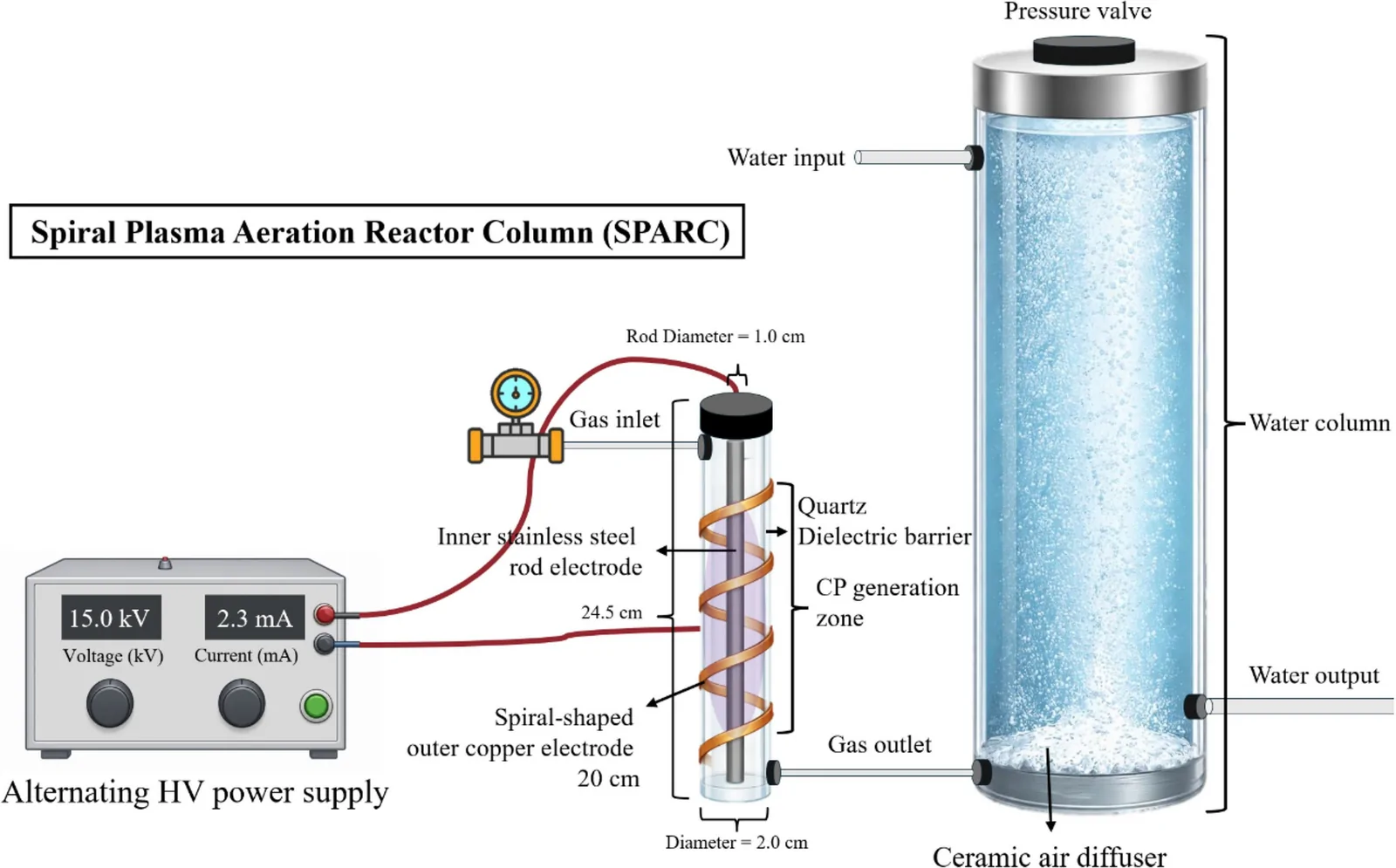
Schematic representation of the Spiral Plasma Aeration Reactor Column (SPARC) used in this study. The system consists of a cold atmospheric plasma generation unit equipped with a spiral-shaped outer copper electrode and a stainless-steel inner electrode, coupled to an air diffuser positioned at the base of a vertical water column. Plasma-generated reactive species are transported into the liquid phase via gas bubbling, enhancing plasma-liquid mass transfer and enabling treatment of larger water volumes. Gas flow rate and electrical input parameters were externally controlled to ensure stable plasma operation

Prior to microbiological assays, operating conditions for the SPARC reactor were systematically optimized to ensure stable CAP generation and reproducible plasma-liquid interactions. All SPARC experiments reported below were conducted under these optimized settings (quartz dielectric tube, stainless‑steel inner rod, spiral copper outer electrode; 15 kV, 2.3 mA; gas flow 2 L·min^−1^; 30 min plasma generation + 30 min only aeration) (Fig. 4). Throughout the experiments, the temperature of the treated liquid was monitored using a VWR® pHenomenal® probe (VWR, USA) and remained between 19.8 and 22.4 °C.

**Fig. 5:**
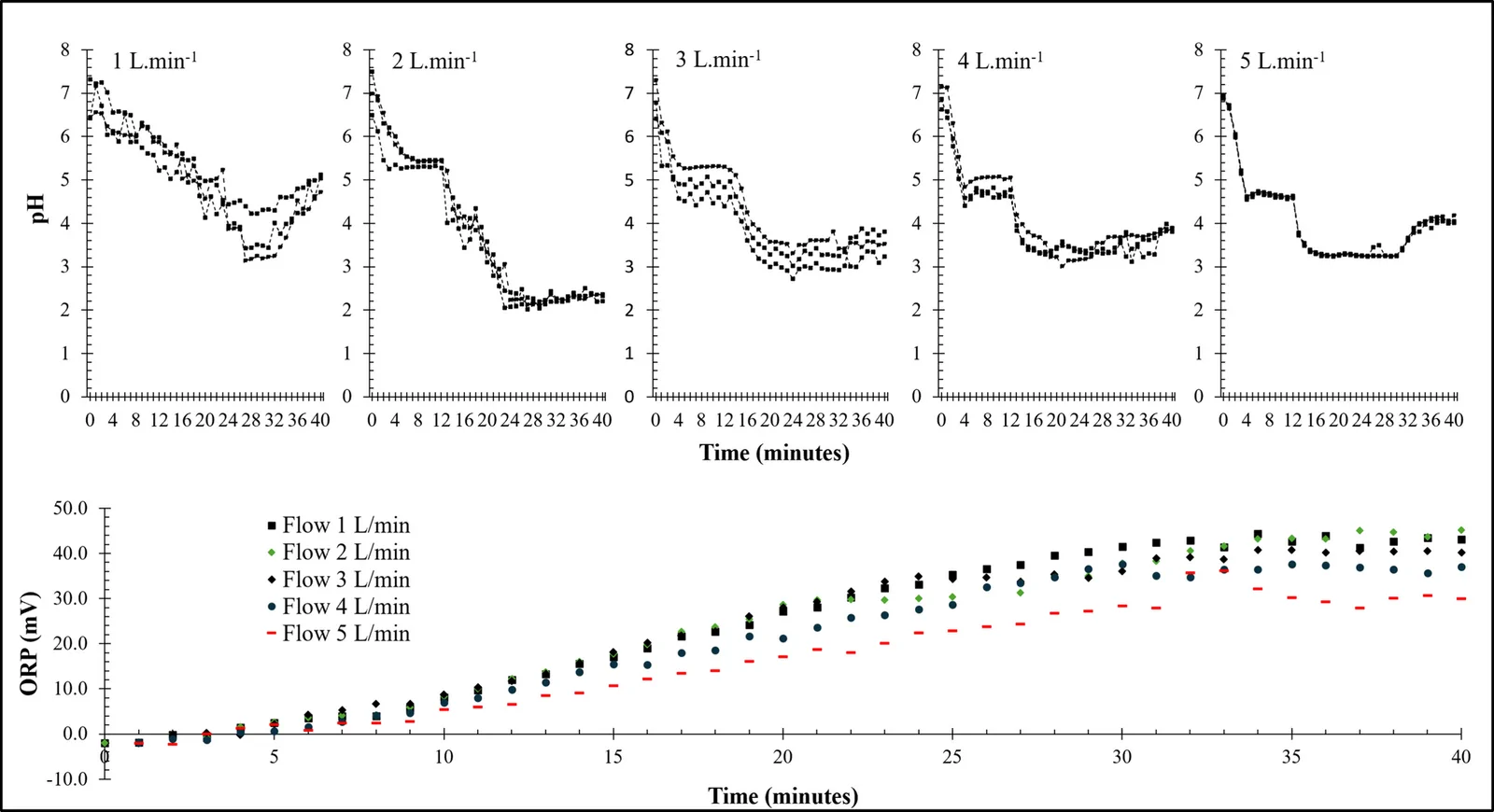
Time evolution of pH and ORP in SPARC-treated distilled water (250 mL). pH (top) and oxidation–reduction potential (ORP, bottom) of sterile distilled water treated with SPARC at 250 mL and 1, 2, 3, 4, and 5 L·min^−1^ gas flow rate, measured during indirect CAP exposure. Values represent mean ± standard deviation of three biological replicates (*n* = 3). Cold plasma and aeration were applied simultaneously for 60 min

Operating conditions for the SPARC were optimized prior to microbiological testing. All experiments were conducted at the lowest electrical settings that consistently produced a visually stable discharge (15 kV, 2.3 mA). The influence of gas flow rate on plasma-induced pH and ORP changes was assessed using 250 mL of distilled water, corresponding to the minimum volume required to fully immerse the air diffuser. Flow rates between 1 and 5 L·min^−1^ were tested (Fig. 5), with plasma applied for 30 min, as pH values approached a plateau within this period.

At a flow rate of 1 L·min^−1^, pH decreased gradually to approximately 3.2–3.5 after 30 min. Increasing the flow rate to 2 L·min^−1^ produced a more pronounced and reproducible acidification, with pH values reaching 2.0–2.3 within 20–25 min before stabilizing. At 3 L·min^−1^, minimum pH values of 2.8–3.1 were reached, but with higher variability between replicates. Although CAP operation remained stable at 4 and 5 L·min^−1^, these conditions led to less pronounced pH decreases. At higher flow rates (> 5 L·min^−1^), CAP generation became unstable and could not be reliably maintained. ORP followed the same general trend, confirming progressive oxidation of the treated liquid under the selected operating conditions. Based on the magnitude, reproducibility, and temporal stability of the pH decrease and ORP increase, a gas flow rate of 2 L·min^−1^ and 30 min of plasma generation were selected for subsequent microbiological experiments.

**Fig. 6:**
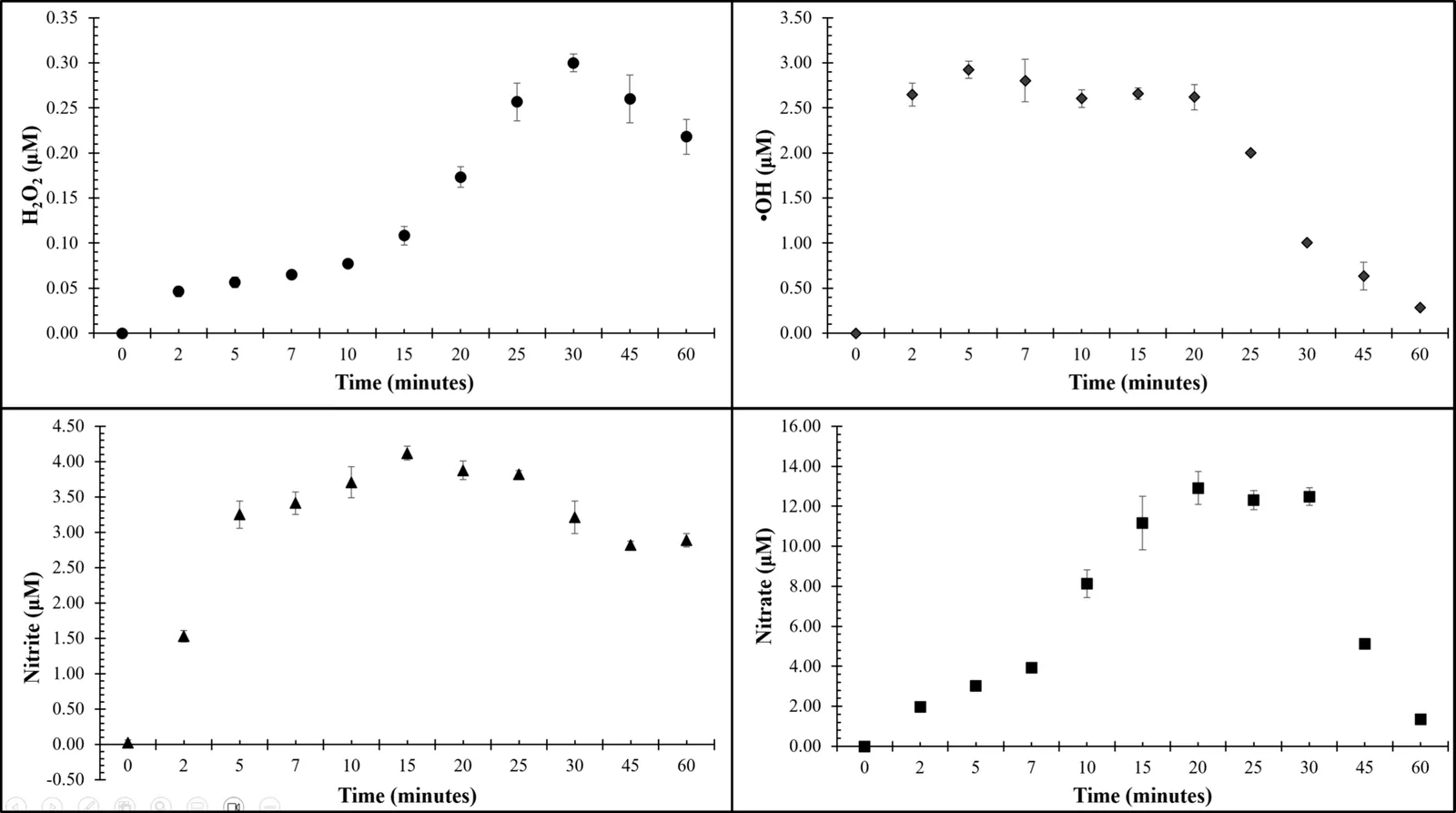
Temporal evolution of hydrogen peroxide (H₂O₂), hydroxyl radicals (*⋅*OH), nitrite (NO₂⁻), and nitrate (NO₃⁻) concentrations in sterile distilled water treated with SPARC at 250 mL and 2 L·min^−1^ gas flow rate. Data are shown as mean ± standard deviation of three biological replicates (*n* = 3). Cold plasma and aeration were applied simultaneously for the first 30 min, after which aeration alone was maintained for an additional 30 min

Under the optimized 250 mL, 2 L·min^−1^ condition, SPARC treatment led to the accumulation of long-lived reactive species in the liquid phase. H₂O₂, nitrite, nitrate, and ⋅OH were all detected and showed time-dependent increases during treatment (Fig. 6), confirming that the optimized indirect configuration generated a reactive plasma-activated liquid environment.

**Fig. 7:**
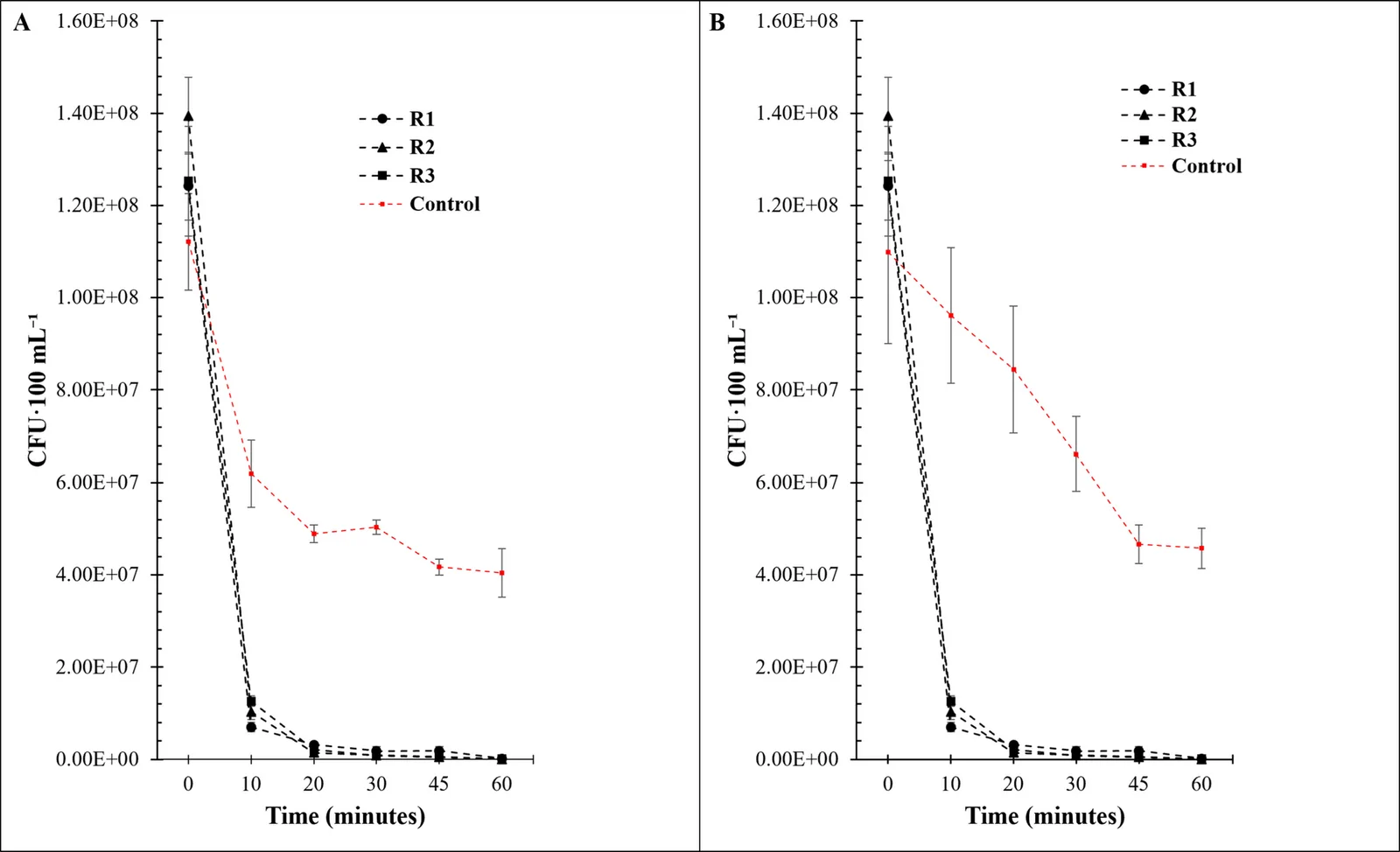
Time-dependent inactivation of *E. coli* (**A**) and *B. subtilis* (**B**) in 250 mL of water treated with an indirect bubble cold plasma system (2 L·min^−1^). Treatment without the bubble diffuser is shown as the control (red dashed line). Values are reported as CFU per 100 mL (mean ± SD)

In 250 mL samples (Fig. 7), the SPARC achieved rapid and efficient bacterial inactivation. For *E. coli*, mean reductions 1‑, 2‑, and ≥ 3‑log₁₀ (92.3 ± 2.2%, 98.3 ± 0.8%, and > 99.9%) were obtained after 10, 20, and 60 min, respectively. For *B. subtilis*, reductions of about 1‑, 2‑, and 3‑log₁₀ (94.0 ± 0.2%, 98.4 ± 0.1%, and 99.7 ± 0.1%) were observed over the same times. In contrast, non‑aerated CAP remained below ≈0.8‑log₁₀ (≤ 60%) reduction after 60 min.

**Fig. 8:**
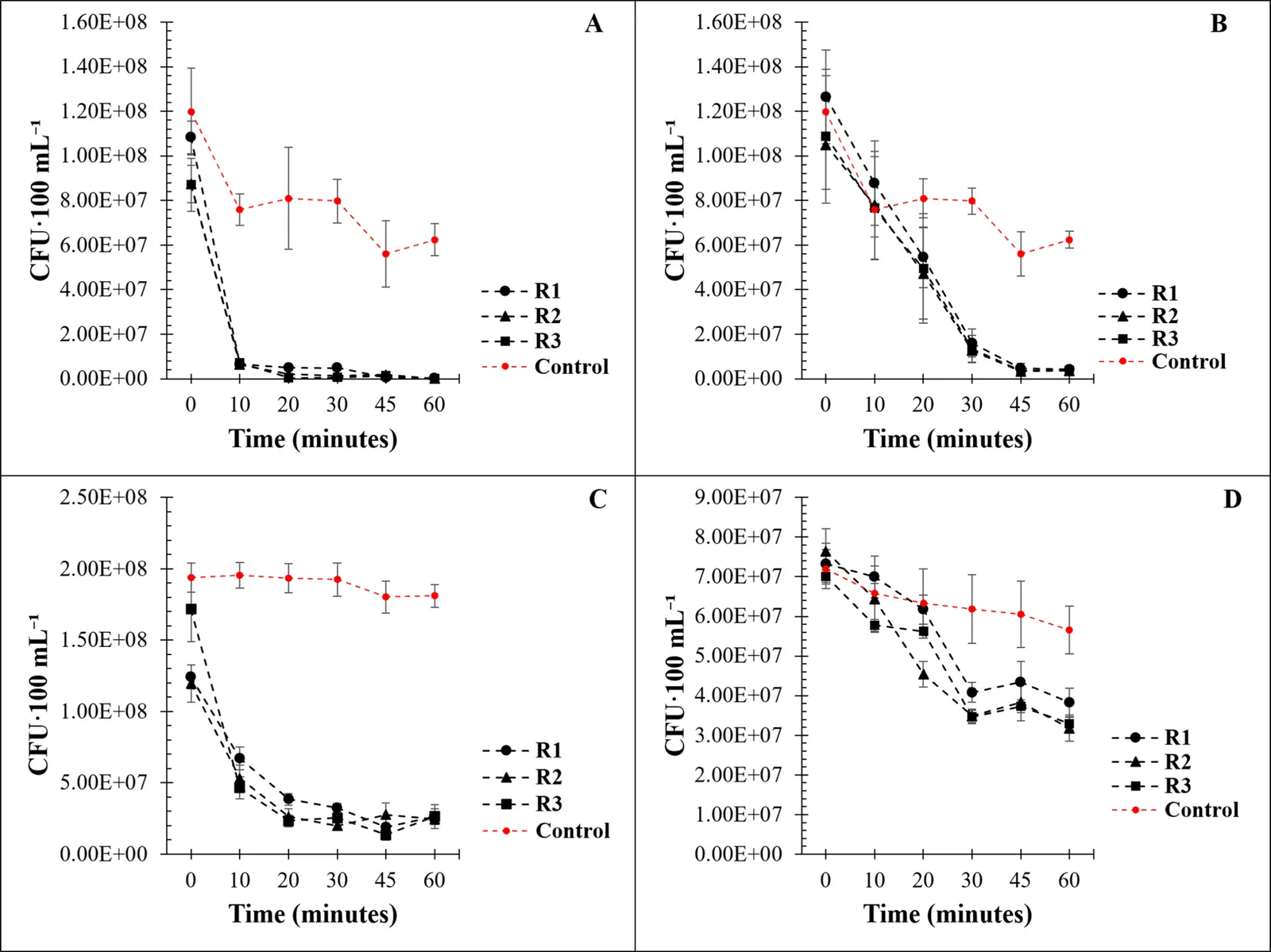
Time-dependent bacterial inactivation under indirect, bubble-assisted cold atmospheric plasma treatment. **A**, **B** The inactivation of *Escherichia coli* (**A**) and *Bacillus subtilis* (**B**) in 500 mL of water and **C**, **D** the inactivation of *E*. *coli* (**C**) and *B*. *subtilis* (**D**) in 1 L of water. Solid black symbols and dashed lines represent independent biological replicates (R1–R3), and the red dashed line corresponds to the control treatment non aerated CAP (no bubble diffuser). Plasma treatment was applied for 30 min at a gas flow rate of 2 L·min^−1^. Results are expressed as CFU per 100 mL (mean ± SD)

SPARC performance was then evaluated at 500 mL and 1000 mL (Fig. 8), and inactivation rates across volumes are summarized in Table 1. In 500 mL, *E. coli* was rapidly inactivated under bubble-assisted plasma conditions, with reductions of 1‑ and 2‑log₁₀ (92.9 ± 1.1% and 97.7 ± 2.0%) after 10 and 30 min, reaching ≥ 3‑log₁₀ (99.9 ± 0.1%) inactivation after 60 min. In the same volume, *B. subtilis* showed slower inactivation kinetics, with reductions of 0.1-, 0.9-, and 1.5-log₁₀ (28.7 ± 2.5%, 87.7 ± 0.3%, and 96.6 ± 0.1%) at 10, 30, and 60 min, respectively. Bubble-assisted CAP remained effective at 1 L, but inactivation kinetics were slower and more variable than at 250–500 mL, particularly for *B. subtilis*, indicating a volume-dependent decline in treatment efficiency.

**Table 1:** Percentage reduction of *Escherichia coli* and *Bacillus subtilis* under indirect cold atmospheric plasma treatment with bubble diffusion (CP + bubbles) and non-aerated CAP (no bubble diffuser) (CP) at treated water volumes of 250 mL, 500 mL, and 1000 mL. Reductions are shown as a function of treatment time. Values are reported as mean ± standard deviation from independent biological replicates (*n* = 3)

	Volume = 250 mL				Volume = 500 mL				Volume = 1000 mL			
Organism	*E. coli*		*B. subtilis*		*E. coli*		*B. subtilis*		*E. coli*		*B. subtilis*	
Time (minutes)	CP + bubbles	CP	CP + bubbles	CP	CP + bubbles	CP	CP + bubbles	CP	CP + bubbles	CP	CP + bubbles	CP
10	92.3 ± 2.2	44.8 ± 1.1	94.0 ± 0.2	12.5 ± 0.2	92.9 ± 1.1	36.6 ± 1.2	28.7 ± 2.5	10.4 ± 1.1	58.1 ± 13.6	0.78 ± 1.1	12.7 ± 7.1	8.6 ± 1.1
20	98.3 ± 0.8	56.4 ± 1.5	96.2 ± 0.2	23.2 ± 0.2	97.4 ± 2.0	32.4 ± 0.9	55.5 ± 1.2	29.4 ± 1.2	77.9 ± 8.6	0.2 ± 0.1	25.4 ± 13.3	12.0 ± 0.9
30	99.1 ± 0.5	55.1 ± 0.8	98.4 ± 0.1	39.8 ± 1.1	97.7 ± 2.0	33.5 ± 0.1.0	87.7 ± 0.3	26.3 ± 0.9	80.9 ± 6.0	0.8 ± 0.1.7	49.8 ± 5.1	14.1 ± 2.1
45	99.3 ± 0.7	62.8 ± 0.7	94.0 ± 4.7	57.5 ± 0.8	98.6 ± 0.8	53.2 ± 2.1	96.5 ± 0.3	24.7 ± 0.5	84.6 ± 7.6	7.1 ± 1.1	45.8 ± 4.6	15.8 ± 1.4
60	99.9 ± 0.1	63.9 ± 0.4	99.7 ± 0.1	58.4 ± 0.6	99.9 ± 0.1	47.9 ± 1.5	96.6 ± 0.1	39.3 ± 1.5	80.9 ± 3.2	6.7 ± 3.4	53.1 ± 5.3	21.4 ± 1.2

Across all tested volumes, bubble-assisted CAP consistently outperformed the non-aerated configuration for both *E. coli* and *B. subtilis*. At 250 and 500 mL, the SPARC achieved ≥ 97–99% inactivation of *E. coli* within 20–30 min and ≥ 96–99% inactivation of *B. subtilis* by 60 min, whereas the non-bubbled reactor generally remained below 60% reduction even after prolonged treatment. These differences indicate that the increased gas–liquid interfacial area in the bubble-assisted configuration substantially enhances the effective dose delivered to the microorganisms.

**Fig. 9:**
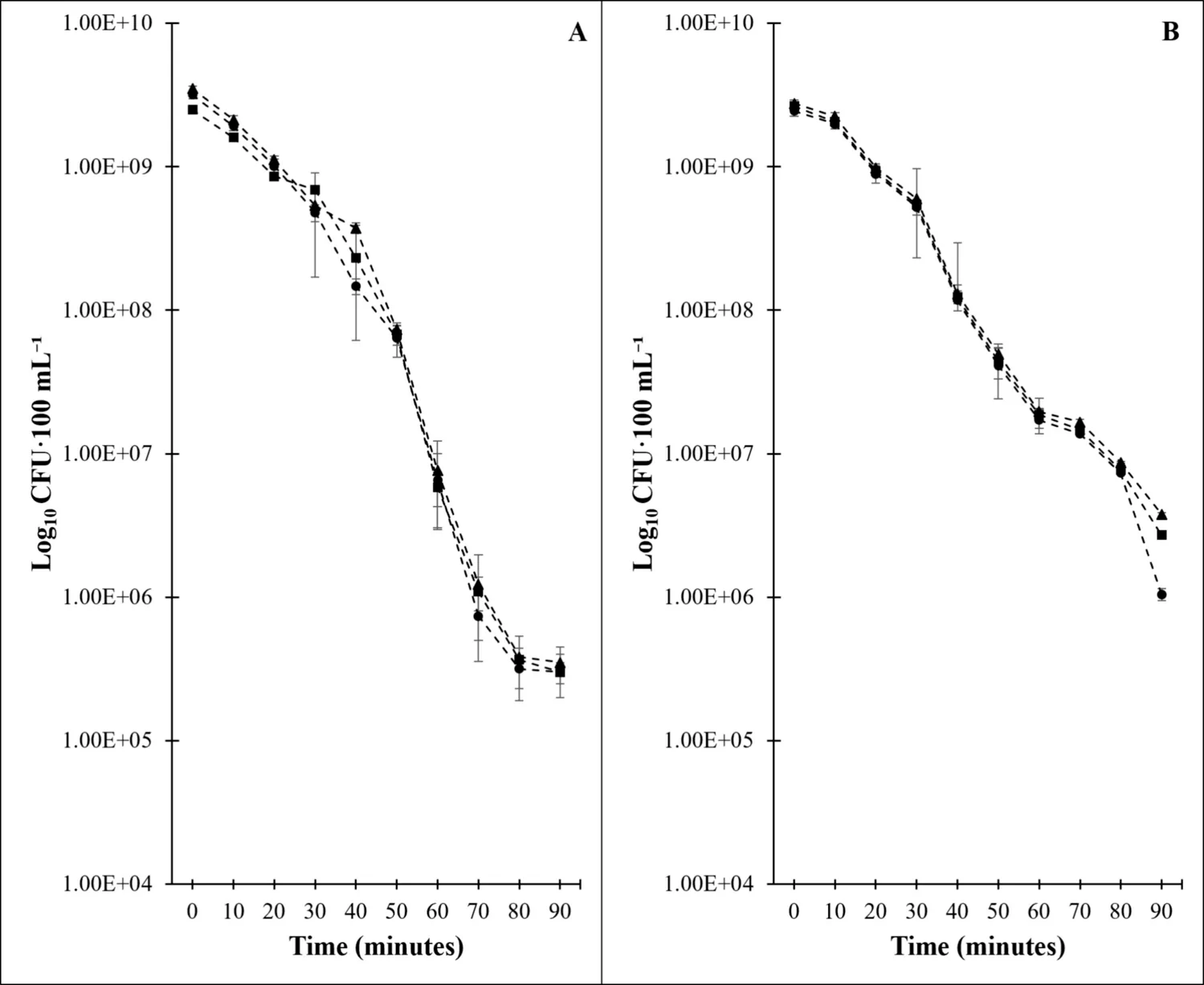
Time-dependent inactivation of *Escherichia coli* (**A**) and *Bacillus subtilis* (**B**) at elevated initial bacterial loads under bubble-assisted cold atmospheric plasma treatment in 1000 mL of water. Experiments were performed using the optimized bubble-assisted configuration with plasma applied for 60 min. Symbols represent independent biological replicates (*n* = 3), and error bars indicate standard deviation within the 3 technical replicates. Results are expressed as log₁₀ (CFU per 100 mL)

The high-load experiments in 1 L samples provided a stringent assessment of the operational limits and practical relevance of the SPARC system. Initial bacterial concentrations on the order of 10^9^ CFU·100 mL^−1^ were used to enable clear quantification of inactivation kinetics over extended treatment times and to test whether the system could achieve meaningful log reductions under heavily contaminated or worst-case conditions (Fig. 9). Plasma generation was applied for 60 min, followed by 30 min of post-treatment monitoring to evaluate potential effects associated with long-lived plasma-generated reactive species.

Under these conditions, the SPARC achieved sustained multi-log₁₀ reductions for both model organisms. Progressive inactivation of *E. coli* and *B. subtilis* yielded approximately a 4-log₁₀ reduction for *E. coli* and a 3.5-log₁₀ reduction for *B. subtilis*. The lower log₁₀ reduction for *B. subtilis* relative to *E. coli* is consistent with the trends observed at lower volumes. Nonetheless, achieving ~ 3.5-log₁₀ inactivation of *B. subtilis* at the litre scale and at extreme initial loads demonstrates the robustness of the bubble-assisted CAP configuration compared with many plasma systems operating at smaller scales.

**Fig. 10:**
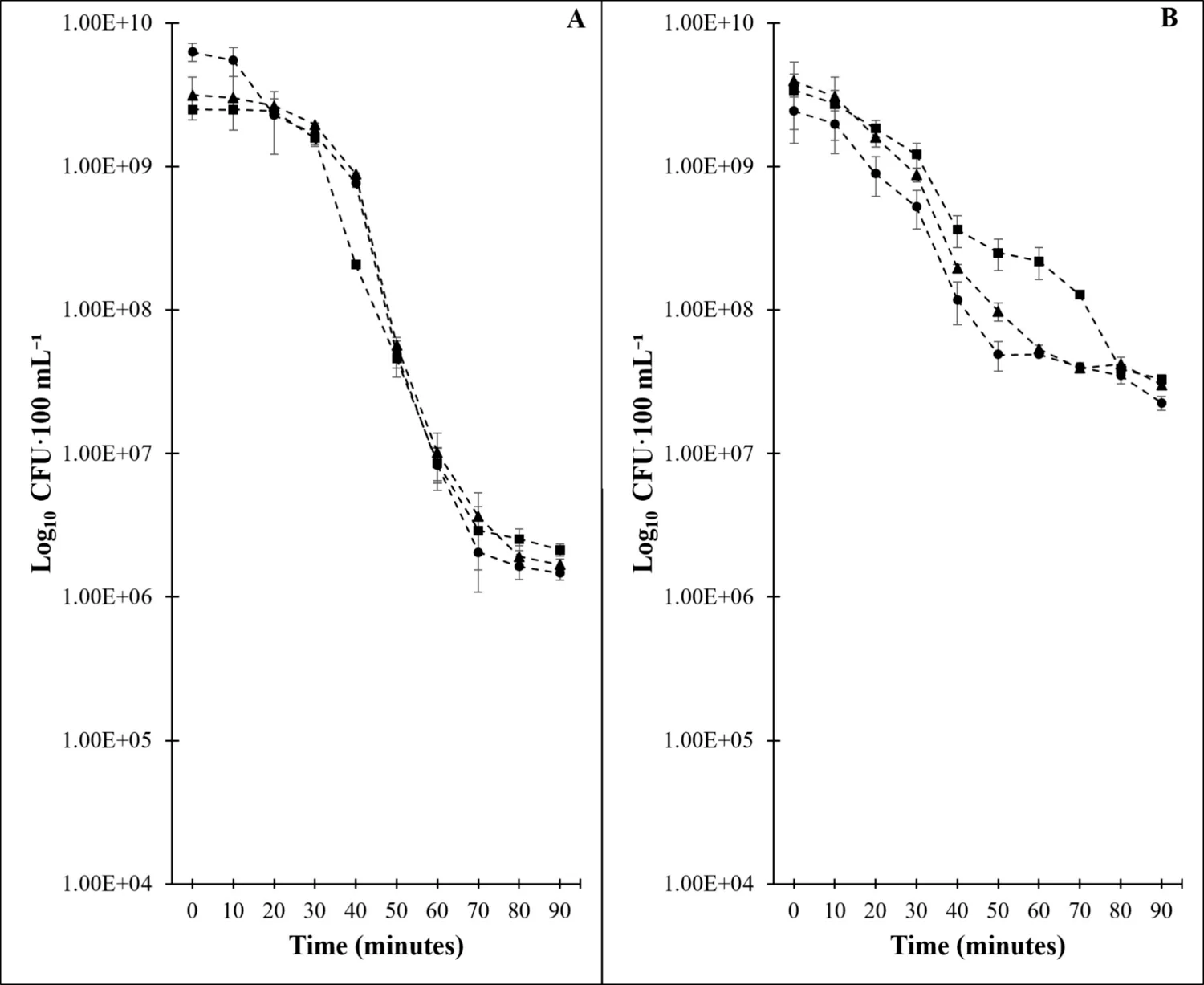
Time-dependent inactivation of *Escherichia coli* (**A**) and *Bacillus subtilis* (**B**) in 1000 mL of complex organic matrix (beef stock–based medium) treated with the SPARC system. Experiments were conducted under the optimized configuration (2 L·min^−1^ gas flow rate), with plasma applied for 60 min and continued monitoring up to 90 min. Symbols represent independent biological replicates (*n* = 3), and error bars indicate standard deviation. Results are expressed as log₁₀ (CFU per 100 mL)

Finally, experiments conducted in a protein-rich organic matrix (beef stock–based medium) were used to simulate wastewater-like conditions (Fig. 10). For *E. coli* (Fig. 10A), inactivation kinetics differed from those observed in distilled water: during the initial treatment phase (0–30 min), reductions proceeded more slowly, indicating partial quenching of reactive species by organic constituents. After approximately 40–50 min of treatment, inactivation rates increased, and by 60 min, *E. coli* counts were reduced by approximately 3 log₁₀ units. Continued inactivation during the post-treatment phase led to final concentrations close to 10^6^ CFU·100 mL^−1^, at 90 min.

For *B. subtilis* (Fig. 10B), inactivation followed a similar but more gradual trend. Early reductions were limited, but sustained exposure resulted in continuous decreases in viable counts over time, reaching approximately 2–3 log₁₀ inactivation by the end of the experiment (Fig. 10).

## Discussion

Direct CAP exposure at the micro-scale produced rapid and complete inactivation of both model organisms (under the limit of quantification of the methods used), consistent with previous reports of highly efficient plasma-liquid interactions under thin-layer conditions (Das et al. 2022; Ezzati et al. 2025). It is widely reported that at the micro-scale, a large portion of the liquid volume remains within a short diffusion distance of the plasma-liquid interface, allowing RONS, UV photons, and transient electric fields to act more uniformly throughout the sample (Bruggeman et al. 2016a; Wong et al. 2023). Under these conditions, concentration gradients of short-lived species are minimized, resulting in near-homogeneous oxidative stress across the bacterial population (Bruggeman et al. 2016a; Wong et al. 2023). The pronounced decrease in inactivation efficiency observed when moving from 200 µL to 100 mL highlights a fundamental limitation of direct CAP exposure, namely that plasma-liquid interactions remain confined to a thin interfacial region and the delivery of reactive species into the bulk liquid becomes increasingly diffusion-limited as volume increases (Foster 2017; Hamza et al. 2023a; Maybin et al. 2024; Pequeno et al. 2026).

The delayed response of *B. subtilis* relative to *E. coli* is consistent with the well-established structural differences between Gram-negative and Gram-positive bacteria. The thicker peptidoglycan layer of *B. subtilis* confers increased resistance to oxidative stress, acting as a physical and chemical barrier that can partially scavenge reactive species before they reach critical intracellular targets (Mai-Prochnow et al. 2016; Xu et al. 2020; Liu et al. 2025). Rapid inactivation of *E. coli* under direct CAP exposure is well documented: both DBD and microjet systems efficiently inactivate *E. coli* in suspended liquid media within seconds to minutes at voltages around 40 kV (Liu et al. 2009; Ziuzina et al. 2012). In contrast, Gram-positive organisms such as *B. subtilis* and their spores consistently show greater resistance in direct-exposure setups, requiring longer exposure times or higher doses for comparable log reductions, which is generally attributed to their thicker peptidoglycan and additional protective structural layers that impede reactive species penetration (Roth et al. 2010; Sun et al. 2012; Pina-Perez et al. 2020).

The optimization of gas flow rate in the SPARC reactor provides mechanistic insight into plasma-liquid mass transfer. Variations in pH and ORP evolution with gas flow rate reflect changes in gas–liquid mass transfer and residence time, which influence the generation, dissolution, and accumulation of plasma-derived reactive species in the liquid phase (Oehmigen et al. 2010; Bruggeman et al. 2016b). Plasma-induced acidification of water and increased ORP has been widely reported and is often used as an indirect indicator of reactive species transfer efficiency and plasma-liquid interaction dynamics (Lukes et al. 2014).

At low flow rates (1 L·min^−1^), the gradual pH decrease suggests limited delivery of plasma-generated reactive nitrogen species to the liquid, likely due to reduced gas throughput and a lower overall flux of reactive species entering the water. Increasing the flow rate to 2 L·min^−1^ suggests an enhanced gas–liquid interfacial area and bubble density, improving the dissolution and accumulation of long-lived plasma-derived species such as nitrite, nitrate, and hydrogen peroxide, which are known to drive plasma-induced acidification through the formation of nitric and nitrous acids. Similar enhancements in plasma-induced pH reduction at intermediate flow rates have been reported in plasma-liquid systems and are attributed to optimized mass transfer and sufficient gas residence time for reactive species solvation (Oehmigen et al. 2010; Lukes et al. 2014; Bruggeman et al. 2016a). At higher flow rates (≥ 3 L·min^−1^), the less pronounced pH decrease and increased variability indicate that reduced gas residence time and intensified gas stripping limit the effective dissolution of reactive species into the liquid phase. This behaviour has been observed previously, where excessive gas flow diminishes reactive species transfer efficiency and suppresses pH reduction due to insufficient contact time at the gas–liquid interface (Marcinauskas et al. 2025). The selection of 2 L·min^−1^ as the operating flow rate is therefore supported both by the observed pH profiles together with the accompanying increase in ORP, which indicates a more oxidizing plasma-treated liquid environment.

Although the RONS assays were performed only under the optimized 250 mL, 2 L·min^−1^ condition, they provide a mechanistic basis for the disinfection results obtained with SPARC. The accumulation of H₂O₂ and nitrogen species is consistent with the sustained inactivation observed during and after plasma exposure, particularly under conditions where post-treatment effects were detected. Together, the pH, ORP, and RONS data indicate that bubble-assisted CAP generates a chemically active liquid phase capable of extending antimicrobial action beyond the discharge period.

The improvement in bacterial inactivation observed in the bubble-assisted configuration underscores the central role of reactor design in governing CAP treatment efficiency (Zeghioud et al. 2020; Vasilev et al. 2024). Importantly, the enhanced performance achieved with the SPARC was obtained without increasing applied voltage, current, or treatment time relative to the non-aerated configuration, indicating that the observed gains arise primarily from improved plasma-liquid mass transfer rather than from increased energy input. This design-driven enhancement enables a larger fraction of the treated volume to be exposed to both short- and long-lived RONS, mitigating the strong concentration gradients that limit direct plasma-liquid contact systems (Gao et al. 2022; Lu et al. 2025; Kuddushi et al. 2025). The performance gap widens at larger volumes and for the more resistant *B. subtilis*, reinforcing that reactor and contactor design become increasingly critical as scale and microbial robustness increase. This also suggests that simple extrapolation of non-aerated CAP systems to litre scales is unlikely to be viable without mass-transfer intensification (Gonçalves et al. 2025; Szulc et al. 2025; Pequeno et al. 2026). In bioprocess terms, bubble enhancement increased the effective dose delivered to the microbial population at fixed electrical input, thereby reducing the specific energy required to achieve a given log reduction and illustrating how contactor design can be exploited to intensify plasma-based disinfection. For bioprocess design, this implies that plasma-based disinfection systems must be dimensioned to meet the requirements of the most resistant relevant targets (for example, spore-forming or Gram-positive organisms) rather than only Gram-negative indicators such as *E. coli* (Maybin et al. 2024; Stapelmann et al. 2024). In this configuration, plasma-liquid interactions likely remain restricted to a thin interfacial region, and the delivery of reactive species into the bulk liquid becomes increasingly diffusion-limited as volume increases (Foster 2017; Hamza et al. 2023a; Pequeno et al. 2026).

The continued decrease in viable counts after plasma shutdown provides evidence for the role of long-lived plasma-generated species, such as hydrogen peroxide, nitrite, nitrate, and secondary reactive nitrogen species, which persist in solution and extend antimicrobial activity beyond the active discharge period (Xu et al. 2020). Similar post-treatment effects have been widely reported in PAW studies and are considered a key advantage of indirect plasma systems (Yoon et al. 2025; Vuković et al. 2025; Guo et al. 2026). The presence of this delayed inactivation phase suggests that the SPARC effectively accumulates chemically active species in the liquid phase, allowing time-integrated microbial stress and providing a reservoir of oxidative capacity that continues to act under storage or distribution conditions.

Experiments performed in a protein-rich organic matrix further highlight both the limitations and the robustness of the SPARC system under chemically complex, application-relevant conditions. Organic matter is known to act as a major sink for plasma-generated reactive species, particularly short-lived radicals and oxidants, through rapid scavenging and competitive reactions (Mai-Prochnow et al. 2021; Hamza et al. 2023b; Moiseienko et al. 2025). This phenomenon has been widely documented for plasma-treated waters and other advanced oxidation processes, where dissolved organic carbon, proteins, and amino acids significantly reduce apparent disinfection kinetics during early treatment stages (Bruggeman et al. 2016b; Attri et al. 2018; Gao et al. 2022). The slower initial inactivation observed in the beef stock matrix is therefore consistent with expectations and reflects a realistic challenge for plasma-based water treatment in wastewater or reuse contexts (Varanasi et al. 2018; Wang et al. 2022; Hamza et al. 2023b). At the same time, the ability of SPARC to achieve multi-log reductions at litre scale in this matrix demonstrates that SPARC can retain significant antimicrobial potency even when a substantial fraction of reactive species is consumed by background organic demand.

Beyond tertiary and quaternary wastewater treatment, the performance of the SPARC system suggests broader applicability in water sanitation contexts requiring decentralized, on-demand disinfection. The ability to achieve multi-log microbial reductions at litre scale without chemical dosing makes bubble-assisted CAP particularly attractive for aquaculture and recirculating water systems (Wang et al. 2024; Thongsopa et al. 2024; Hashempour-baltork et al. 2024; Balazinski et al. 2025), where residual disinfectants can negatively affect aquatic organisms and biofilters. Similarly, clinical and laboratory wastewater streams, which often contain high loads of ARB, could benefit from localized CAP treatment to reduce microbial risks prior to discharge (Gururani et al. 2021; Hamid et al. 2024). The system also shows potential for decentralized water treatment and emergency sanitation, as CAP relies only on electricity and ambient air and avoids the storage and handling of hazardous chemicals. Its modularity and intermittent operation further support adaptation to point-of-use or small-community applications (Reed et al. 2013; Bastable and Russell 2013; Yates et al. 2018).

Despite these advantages, important limitations remain. Although bubble-assisted CAP substantially improved plasma-liquid mass transfer, treatment efficiency remained volume-dependent, with reduced performance at 1 L compared to smaller volumes. Water matrix complexity significantly affected inactivation kinetics, as demonstrated by slower initial reductions in the protein-rich organic medium. Organic scavenging of plasma-generated reactive species is a known challenge for CAP and other advanced oxidation processes, highlighting the need for systematic evaluation in real wastewaters with varying organic loads and ionic compositions.

RONS, together with pH and ORP, under the optimized SPARC operating condition, provide direct mechanistic support for the observed inactivation and post-treatment effects. Dissolved ozone, conductivity, and peroxynitrite-related chemistry were not quantified in this study and remain valuable additions for future work.

Recent reviews emphasise that plasma-liquid chemistry is governed by a complex, coupled reaction network in which the “effective dose” arises from a dynamic mixture of short‑ and long‑lived species rather than a single molecular marker, and that reported RONS concentrations vary widely across devices, operating conditions, and matrices. In this proof‑of‑concept study, we therefore prioritized microbiological endpoints across volumes and water matrices, while leaving high‑resolution RONS speciation and detailed chemical dosimetry to future work (Bruggeman et al. 2016b). This study did not include detailed plasma diagnostics such as optical emission spectroscopy, current–voltage waveform analysis, Lissajous-figure-based discharge power determination, or discharge imaging. Such measurements would provide valuable mechanistic insight into discharge behaviour and plasma stability but were beyond the scope of the present bioprocess-oriented evaluation, which prioritized inactivation performance and reactor-level mass transfer over fundamental discharge characterization.

While CAP is often described as energy-efficient at the laboratory scale (Gavahian et al. 2024), quantitative energy-per-log-reduction assessments are essential to benchmark plasma-based bioprocesses against established tertiary and quaternary disinfection technologies such as UV and ozonation. In this study, SPARC operated at fixed electrical conditions (34.5 W, 15 kV, 2.3 mA) throughout all experiments, enabling direct comparison of specific energy input across volumes and conditions. At 250 mL and 30 min treatment (typical for > 99% *E. coli* inactivation), energy consumption was 60 kWh·m^−3^; at 1 L and 60 min (≈4-log *E. coli* inactivation), it was 30 kWh·m^−3^, or ≈7.5 kWh·log₁₀^−1^·m^−3^. Energy consumption is reported as the electrical demand of the plasma discharge in the laboratory prototype. These values are on the order of pilot-scale UV disinfection (10–50 kWh·m^−3^ for 4-log *E. coli* in clear water) and electrochemical advanced oxidation processes (20–100 kWh·m^−3^), but higher than optimized ozonation (1–10 kWh·m^−3^). Bubble enhancement reduced energy per log reduction by enabling higher inactivation at fixed power and time compared with non-aerated CAP, highlighting the value of reactor design for energy efficiency. Future optimization should target lower power inputs, pulsed operation, and continuous-flow configurations to approach industrial benchmarks (< 5 kWh·m^−3^).

Future work should also extend beyond vegetative bacteria to include viruses, spores, protozoa, ARGs, and antibiotic residues, in order to align bubble-assisted CAP with emerging regulatory frameworks and the broader One Health agenda.

## Conclusions

The bubble‑enhanced SPARC reactor addresses a key bottleneck in applying CAP as an environmental bioprocess for water disinfection by intensifying plasma-liquid mass transfer and extending effective treatment from microlitre to sub‑litre and litre scales. Direct DBD exposure achieved very rapid, complete inactivation of *E. coli* and *B. subtilis* at 200 µL, but disinfection efficiency decreased markedly at 100 mL, confirming that conventional surface‑contact CAP is intrinsically volume‑limited by the thin interfacial reaction zone and diffusion‑controlled delivery of reactive species.

By integrating CAP with controlled gas bubbling, the SPARC system substantially improved the distribution and utilization of plasma-generated RONS, achieving ≥ 99–99.9% inactivation of both model bacteria at 250–500 mL, with significantly higher reductions than the non-aerated configuration operated at the same voltage, current, and treatment time. These gains demonstrate that reactor design and contactor geometry can be at least as important as plasma source characteristics in determining CAP performance at increasing volumes. The results show that SPARC remains effective at larger working volumes, but treatment efficiency decreases as volume increases, with the clearest slowdown observed for *B. subtilis* at 1 L. This suggests that reactor design and plasma-liquid interaction remain important factors for scale-up.

High-load experiments at 1 L confirmed that SPARC maintains meaningful disinfection capacity under worst-case conditions, with ≈4-log inactivation of *E. coli* and ≈3–3.5-log inactivation of *B. subtilis* from initial densities of ≈10^9^ CFU·100 mL^−1^, and with continued post-plasma inactivation indicative of PAW-type long-lived RONS effects. Tests in a protein-rich organic matrix further showed that organic load slows early-stage kinetics but does not eliminate disinfection, aligning with the known radical-scavenging role of dissolved organic matter and emphasizing the need to account for matrix effects in wastewater and reuse applications. The SPARC concept illustrates how bubble‑enhanced DBD design can be used as an integrated bioprocess platform to connect CAP reactor/contact geometry, multiscale bacterial inactivation, and specific energy benchmarking at sub‑litre and litre volumes.

## Data Availability

All data supporting the findings of this study are available within the paper.
